# Clinical treatment of tubal pregnancy with methotrexate: a comparison of intramuscular and intravenous routes in a randomized clinical trial

**DOI:** 10.61622/rbgo/2026rbgo78

**Published:** 2026-10-30

**Authors:** Paula Vieira de Mello, Edward Araujo Júnior, Eduardo Moreira Nader, Beatriz Bussi Rosolen, Julio Elito, Antonio Braga, Barbara Milena Marciniak, Eduardo Schunemann, Rafael Frederico Bruns

**Affiliations:** 1 Universidade Federal do Paraná Curitiba PR Brazil Universidade Federal do Paraná, Curitiba, PR, Brazil.; 2 Universidade Federal de São Paulo Escola Paulista de Medicina São Paulo SP Brazil Escola Paulista de Medicina- Universidade Federal de São Paulo, São Paulo, SP, Brazil.; 3 Universidade Municipal de São Caetano do Sul São Caetano do Sul SP Brazil Universidade Municipal de São Caetano do Sul, São Caetano do Sul, SP, Brazil.; 4 Universidade Federal do Rio de Janeiro Rio de Janeiro RJ Brazil Universidade Federal do Rio de Janeiro, Rio de Janeiro, RJ, Brazil; 5 Universidade Federal do Estado do Rio de Janeiro Rio de Janeiro RJ Brazil Universidade Federal do Estado do Rio de Janeiro, Rio de Janeiro, RJ, Brazil.; 6 Universidade de Vassouras Vassouras RJ Brazil Universidade de Vassouras, Vassouras, RJ, Brazil.

**Keywords:** Pregnancy, tubal, Methotrexate, Conservative treatment, Injections intravenous

## Abstract

**Objective:**

To compare the intravenous and intramuscular administration routes of methotrexate in the clinical treatment of tubal pregnancy.

**Methods:**

A prospective randomized clinical trial was conducted, including women with an indication for medical management of tubal pregnancy, comparing the use of methotrexate via intravenous and intramuscular routes (50 mg/m² of body surface area). The analyzed outcome was treatment success (good outcome), defined as a 15% decrease in β-hCG levels between days 1 and 7. Treatment failure was defined as the need for surgical intervention due to suspicion or confirmation of ruptured tubal pregnancy.

**Results:**

A total of 107 cases of tubal pregnancy were included, divided into the intravenous group (n = 48) and the intramuscular group (n = 59). The mean age of the women was 28.1 years (range, 18–40). The mean mass size was 21.8 mm (range, 7–49 mm), and the median [Q1–Q3] initial serum β-hCG value was 1361,15 mU/mL (584,58–2382,45). No significant differences were found between the groups regarding treatment success or failure outcomes, nor in adverse effects. The treatment success rates in the intravenous and intramuscular groups were 74.6% and 79.2%, respectively.

**Conclusion:**

The intravenous and intramuscular routes for the clinical treatment of tubal pregnancy with methotrexate were equivalent in treatment success outcomes, with no difference observed in the reduction of serum β-hCG levels.

**Brazilian Clinical Trials Registry (ReBEC):**

# RBR-7nkf8tn

## Introduction

Tubal ectopic pregnancy remains a leading cause of maternal morbidity and mortality in the first trimester, accounting for up to 1-2% of all pregnancies globally.^(1,2)^ The severity of this condition, closely tracked through national mortality and health registries, requires prompt and effective medical intervention. For hemodynamically stable patients, medical management with methotrexate (MTX) has become the gold standard, offering a safe alternative that avoids the morbidity of surgical intervention. The established protocol typically involves a single intramuscular (IM) dose of 50 mg/m², with careful monitoring of serum human chorionic gonadotropin (β-hCG) until complete clinical resolution.^(3,4)^

Despite the high success rates of the standard IM regimen, optimizing pharmacological delivery remains a clinical priority. As a folate antagonist, MTX interferes with DNA synthesis in rapidly dividing trophoblastic cells. Its maximal therapeutic efficacy is closely tied to achieving a high peak serum concentration.^(5,6)^ Intravenous (IV) administration can theoretically achieve higher and faster serum peaks compared to the IM route, a pharmacokinetic principle widely applied in oncology but underexplored in obstetrics.^(7)^ Despite these potential benefits, direct comparative data for tubal pregnancies is lacking. To our knowledge, this is the first randomized clinical trial to directly compare IV and IM MTX for tubal ectopic pregnancy. A comprehensive search of the PubMed/MEDLINE database up to June 2026, using the terms "methotrexate," "intravenous," "intramuscular," and "tubal pregnancy," yielded no previous randomized controlled trials comparing these two administration routes.

Therefore, the primary objective of this randomized clinical trial was to compare the efficacy of the intravenous versus the intramuscular route for the clinical treatment of unruptured tubal ectopic pregnancy. As a secondary objective, we aimed to evaluate and compare the occurrence of MTX-related adverse effects between the two groups.

## Methods

This was an open-label, prospective, randomized interventional trial evaluating medical treatment in women with unruptured tubal ectopic pregnancy between September 2022 and July 2025. The diagnostic criteria were a positive serum β-hCG above the laboratory reference threshold (>2.3 mU/mL), together with transvaginal ultrasonography findings suggestive of tubal pregnancy (image compatible with a gestational sac or a complex mass with a hypoechoic area in the adnexal region). Due to the distinct nature of the administration routes (bolus intravenous injection vs. undiluted intramuscular injection), participants and care providers were not blinded to the intervention (open-label design).

Methotrexate was administered at a dose of 50 mg/m² body surface area, regardless of allocation group (standard single-dose regimen). Consecutive women presenting to the Emergency Department of the Maternity Unit of the Clinics Hospital, *Universidade Federal do Paraná* (UFPR), with a diagnosis of tubal ectopic pregnancy were invited to participate. Participants were randomized into two groups using permuted block randomization to ensure an equal distribution of participants between the two intervention arms.

A computer-generated block randomization sequence was created using a block size of four and a 1:1 allocation ratio. Within each block, four treatment assignments (two intervention and two control) were arranged in a random order. The control group received intramuscular methotrexate (usual route of administration), and the intervention group received the same dose of methotrexate intravenously. After enrollment, the attending physician contacted the study team, who assigned the participant according to the next available allocation in the sequence and informed the physician of the assigned study group.

Before methotrexate administration, women underwent laboratory testing, including complete blood count, renal function, and liver function, to screen for clinical contraindications to methotrexate use. Intravenous administration was performed as a bolus, diluted in 50–100 mL of normal saline, whereas intramuscular administration was undiluted. According to the routine of the Department of Obstetrics and Gynecology at the Clinics Hospital – UFPR, medical treatment with methotrexate was performed in the maternity emergency unit without the need for hospital admission.

All women were instructed to return on days 4, 7, and 14 after methotrexate administration for follow-up, including symptom assessment and serum β-hCG measurement. They also received counseling on warning signs requiring urgent reassessment (severe abdominal pain, symptoms of hypotension or presyncope, and increased vaginal bleeding) and on post-treatment precautions, such as avoiding intense physical exertion and sun exposure. Adverse effects were monitored non-systematically through patient interviews during follow-up visits on days 4, 7, and 14, and through spontaneous reporting. The primary outcome was treatment success (favorable evolution), defined as a ≥15% decline in serum β-hCG between days 1 and 7. Treatment failure was defined as the need for surgical intervention due to suspected or confirmed ruptured tubal ectopic pregnancy. In cases requiring a second dose of methotrexate to achieve the expected decline in serum β-hCG, the additional dose was administered via the same route initially allocated. These cases were classified as successful outcomes if the expected β-hCG decline occurred after the second dose.

Inclusion criteria were: hemodynamically stable women with a diagnosis of tubal ectopic pregnancy, serum β-hCG < 5,000 mU/mL, absence of embryonic cardiac activity, and an intact adnexal mass < 4 cm on transvaginal ultrasonography. Exclusion criteria were: age < 18 years and any contraindication to medical management of tubal ectopic pregnancy with methotrexate. The following clinical data were collected: date of last menstrual period, serial serum β-hCG values during follow-up, and final outcome (complete resolution, with or without the need for a second dose, or need for surgical management). Participants excluded post-randomization due to subsequent findings of exclusion criteria or loss to follow-up were analyzed using a per-protocol approach, and these losses were documented in the CONSORT flow diagram.

Data were analyzed using Stata/SE version 14.1 (StataCorp LP, College Station, TX, USA). Quantitative variables were described as median and interquartile range (IQR), while categorical variables were described as frequency and percentage. Comparisons between groups were performed using the Student's t-test or the Mann–Whitney test for quantitative variables, and Fisher's exact test for categorical variables. The primary analysis was a comparison of proportions (treatment success vs. failure) between the two routes of administration using Fisher's exact test. Effect estimates were reported as risk ratios (RR) with 95% confidence intervals (CI). As a secondary analysis, the longitudinal evolution of serum β-hCG levels (at baseline, days 4, 7, and 14) was evaluated using generalized estimating equations (GEE) to model population-averaged trends, assuming an exchangeable correlation structure. Log-transformed β-hCG values were used to account for their non-normal distribution. The normality assumption for other quantitative variables was assessed using the Kolmogorov–Smirnov test. A p-value < 0.05 was considered statistically significant.

The study was approved by the Research Ethics Committee of the UFPR under protocol number CAAE: 12014019.4.0000.0096 and approval number 5.370.501, and all participants provided written informed consent. The trial was registered in the Brazilian Clinical Trials Registry (ReBEC) under the identifier RBR-7nkf8tn in May 2022.

## Results

During the study period, data from 107 cases of tubal ectopic pregnancy in 106 women were analyzed, as one woman experienced two tubal ectopic pregnancies. Of the 139 initially recruited patients, 13 were excluded after randomization because they met exclusion criteria (4 due to serum β-hCG > 5,000 mU/mL, 1 due to β-hCG < 2.3 mU/mL, 5 due to adnexal mass size > 4 cm, 2 due to non-tubal ectopic pregnancies, and 1 due to age 17 years), and 19 were excluded due to loss to follow-up after allocation (6 in the intramuscular group and 13 in the intravenous group) (Figure 1).

**Figure 1:**
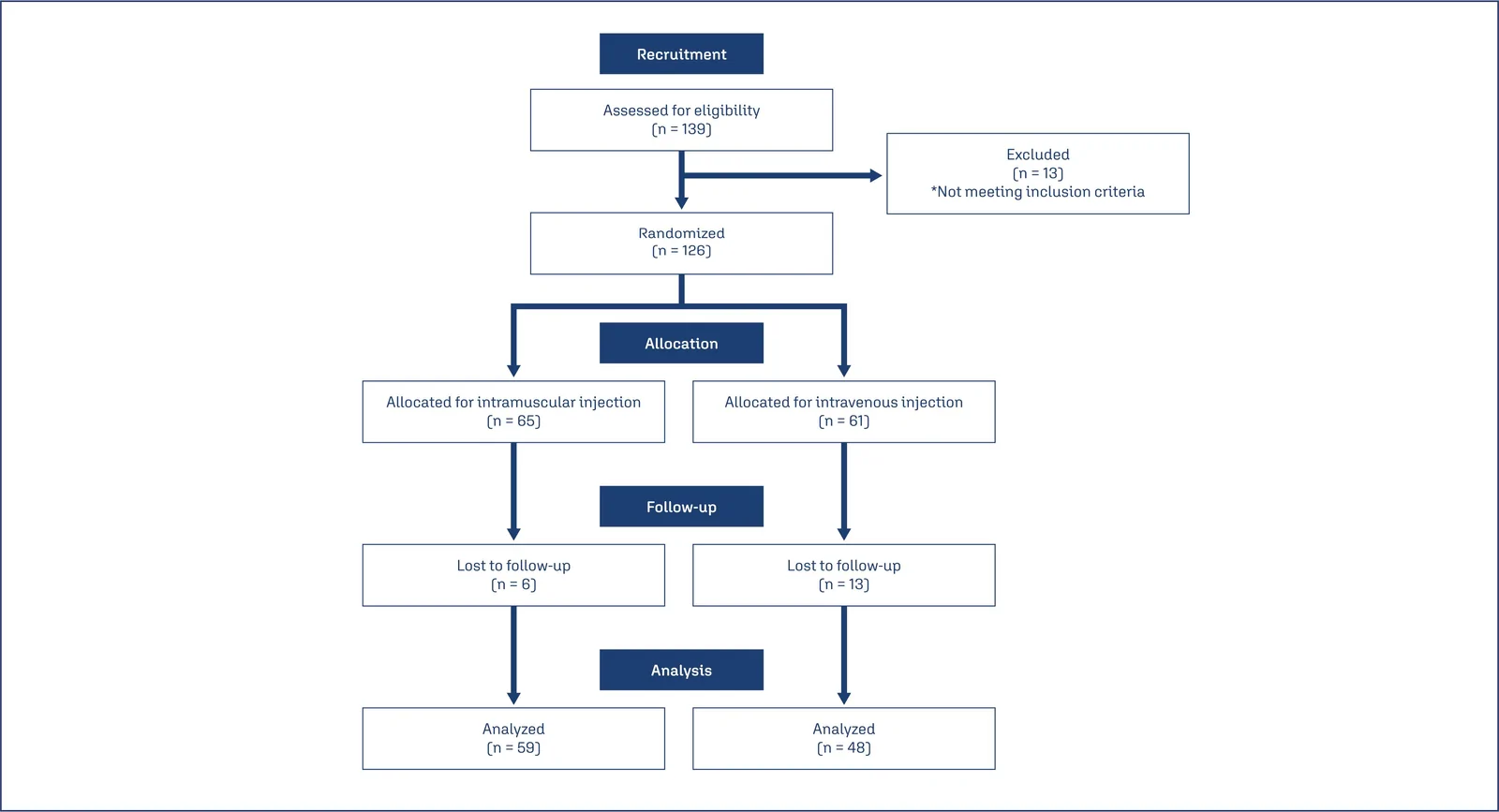
CONSORT flowchart

The mean ± SD age of participants was 28.1 ± 5.1 years (range, 18–40). The mean ± SD mass size was 21.8 ± 7.2 mm (range, 7–49 mm), and the median [Q1–Q3] initial serum β-hCG value was 1361,15 mU/mL (584,58–2382,45). Regarding obstetric history, 71 women (67%) were primigravidas at the time of the tubal ectopic pregnancy diagnosis, and 12 (11.3%) had a history of previous tubal ectopic pregnancy. Most women (79.8%) received methotrexate on the same day as their emergency department visit, with the longest interval being 11 days in one case. There was a significant difference in age between groups according to route of administration: women in the intravenous group had a mean ± SD age of 26.5 ± 4.6 years, whereas those in the intramuscular group had a mean ± SD age of 29.4 ± 5.3 years. The groups were homogeneous with respect to mass size and baseline serum β-hCG levels (Table 1).

**Table 1:** Clinical and ultrasonographic characteristics of women undergoing clinical treatment with methotrexate for tubal pregnancy

Variables	Route of administration	n	Median (min–max) IQR	p-value*
Age (years)	Intramuscular	59	29 (19; 40) IQR=7	
	Intravenous	48	26 (18; 39) IQR=5	0.003
Mass size (largest axis in millimeters)	Intramuscular	57	21 (7; 40) IQR=11	
	Intravenous	47	22 (13; 49) IQR=10	0.404
Initial serum β-hCG value (mU/mL)	Intramuscular	59	1415 (97; 4975) IQR=1694	
	Intravenous	48	1149 (209; 4877) IQR=1858	0.441

* Student's t-test or Mann– Whitney test; IQR: interquartile range.

Treatment success was achieved in 38 of 48 patients (79.2%) in the intravenous methotrexate group and 44 of 59 patients (74.6%) in the intramuscular group. There was no statistically significant difference between treatment groups (Fisher's exact test, p = 0.650). The corresponding risk ratio (RR) for treatment success was 1.06 (95% confidence interval [CI], 0.86–1.31), indicating comparable effectiveness between the two treatment strategies. In both treatment groups, β-hCG concentrations declined significantly throughout follow-up. Although β-hCG values were lower in the intravenous group at several time points, the GEE analysis demonstrated that the overall decline over time was similar between groups, with no significant treatment-by-time interaction, indicating comparable longitudinal response patterns following intravenous and intramuscular methotrexate administration. There was a trend toward higher serum β-hCG values on days 4 and 7 in the intramuscular group. On day 7, the median β-hCG level was 403 mU/mL in the intravenous group and 716 mU/mL in the intramuscular group (p = 0.057). Regarding β-hCG reductions, only the decline from day 4 to day 7 differed significantly, being more pronounced in the intramuscular group (Table 2).

**Table 2:** Evaluation of serum βHCG follow-up after clinical treatment with methotrexate in women with tubal pregnancy by intervention group

Variables	Route of administration	n	Median (min–max) IQR	p-value*
Initial serum β-hCG value (mU/mL)	Intramuscular	59	1415 (97; 4975) IQR=1694	
	Intravenous	48	1149 (209; 4877) IQR=1858	0.441
Serum β-hCG value on day 4 (mU/mL)	Intramuscular	55	1292 (20; 6113) IQR=2345	
	Intravenous	44	588 (5; 7429) IQR=1343	0.076
Serum β-hCG value on day 7 (mU/mL)	Intramuscular	55	716 (30; 6323) IQR=2650	
	Intravenous	38	403 (3; 2431) IQR=1211	0.057
Serum β-hCG value on day 14 (mU/mL)	Intramuscular	49	237 (3; 4377) IQR=584	
	Intravenous	34	112 (2; 1159) IQR=323	0.157
Initial reduction – day 4 (mU/mL)	Intramuscular	55	50 (-4480; 2082) IQR=583	
	Intravenous	44	250 (-3264; 4000) IQR=600	0.078
Initial reduction – day 7 (mU/mL)	Intramuscular	55	419 (-3814; 2499) IQR=797	
	Intravenous	38	402 (-836; 4250) IQR=771	0.876
Initial reduction – day 14 (mU/mL)	Intramuscular	49	1095 (-1257; 4903) IQR=1275	
	Intravenous	34	677 (56; 4737) IQR=1353	0.746
Reduction day 4 – day 7 (mU/mL)	Intramuscular	54	265 (-2090; 2548) IQR=515	
	Intravenous	38	123 (-116; 1185) IQR=257	0.032

* t-Student or Mann-Whitney; IQR: interquartile range

To compare the intramuscular and intravenous groups in terms of β-hCG evolution, a multilevel mixed linear regression model was fitted, and no statistically significant difference was observed between routes of administration (p = 0.501) (Figure 2).

**Figure 2:**
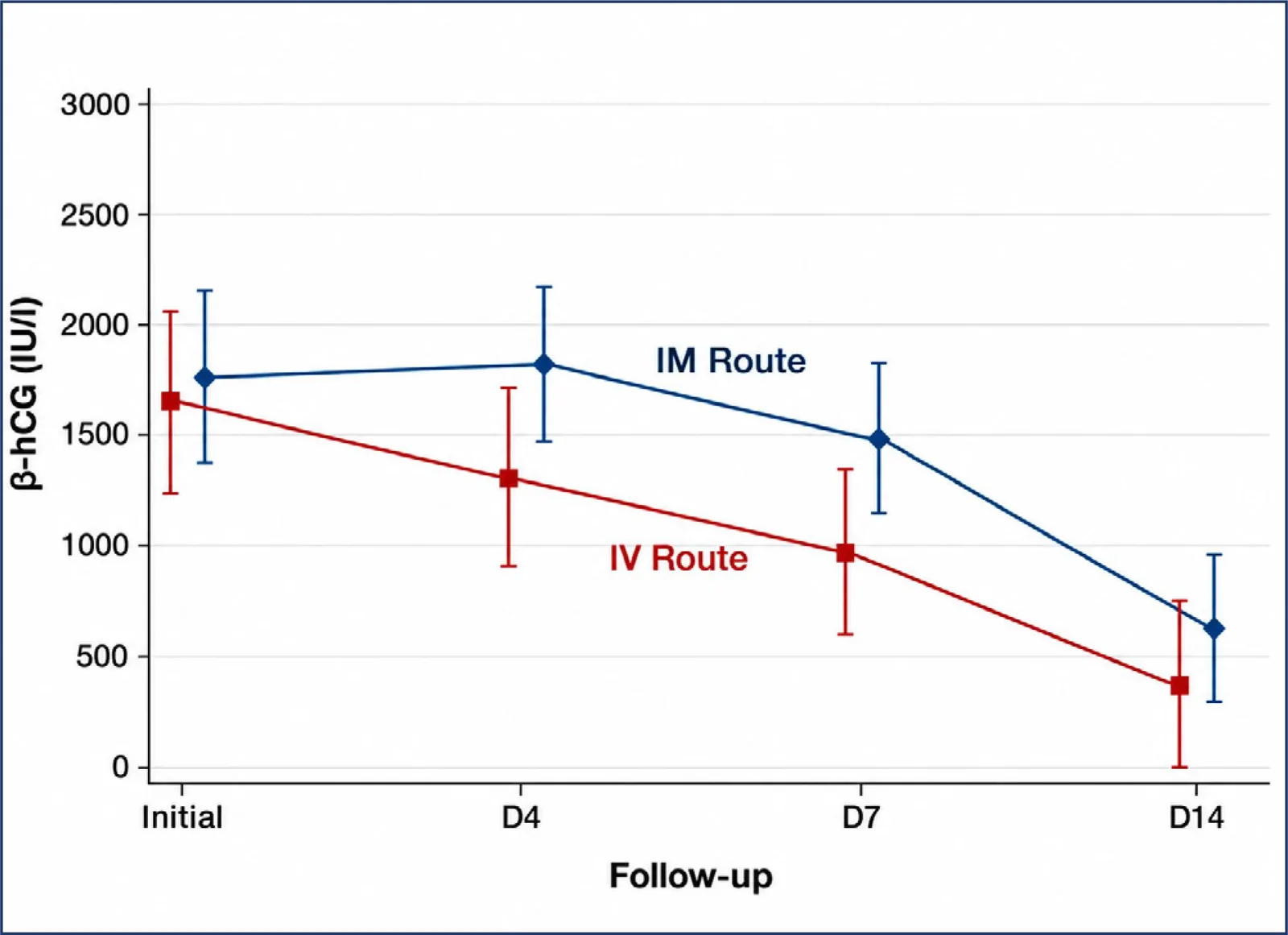
Estimated serum β-hCG values between initial and 14 days in intravenous and intramuscular groups (p=0.763)

Overall, 82 cases (76.6%; 95% confidence interval, 68.0%–83.9%) had a favorable outcome, and surgical treatment was required in 25 cases (23.4%). There was no statistically significant difference in outcomes between the intramuscular and intravenous groups (p = 0.650). A second dose of methotrexate was required in 7 women (6.5%). There was no significant difference in adnexal mass size at diagnosis between women who had successful medical treatment and those who progressed to tubal rupture after methotrexate therapy. Serum β-hCG values were higher in cases of tubal rupture from the initial assessment and remained significantly higher on days 4, 7, and 14. Regarding β-hCG reductions, women with favorable outcomes showed a more marked decline from baseline to day 4 and from baseline to day 7, with statistically significant differences (p = 0.014 and p = 0.008, respectively), whereas reductions from baseline to day 14 and from day 4 to day 7 did not differ between groups (Table 3).

**Table 3:** Evaluation of outcomes according to mass size and serum β-hCG follow-up after clinical treatment with methotrexate in women with tubal pregnancy

Variables	Outcome	n	Median (min–max) IQR	p-value*
Mass size (largest axis in mm)	Good outcome	79	21 (7; 40) IQR=9	
	Tubal rupture	25	22 (13; 49) IQR=10	0.231
Initial serum β-hCG value (mU/mL)	Good outcome	82	1227 (97; 4975) IQR=1494	
	Tubal rupture	25	2361 (121; 4877) IQR=2573	0.014
Serum β-hCG value on day 4 (mU/mL)	Good outcome	81	727 (5; 6113) IQR=1440	
	Tubal rupture	18	2453 (14; 7429) IQR=3768	0.012
Serum β-hCG value on day 7 (mU/mL)	Good outcome	80	447 (3; 5447) IQR=1236	
	Tubal rupture	13	2862 (212; 6323) IQR=2428	<0.001
Serum β-hCG value on day 14 (mU/mL)	Good outcome	77	113 (2; 4377) IQR=349	
	Tubal rupture	6	1642 (372; 3816) IQR=1089	<0.001
Initial reduction – day 4 (mU/mL)	Good outcome	81	130 (-4480; 4000) IQR=467	
	Tubal rupture	18	-187 (-3264; 3208) IQR=1133	0.014
Initial reduction – day 7 (mU/mL)	Good outcome	80	444 (-3814; 4250) IQR=720	
	Tubal rupture	13	-91 (-3753; 925) IQR=1260	0.008
Initial reduction – day 14 (mU/mL)	Good outcome	77	1026 (-1084; 4903) IQR=1283	
	Tubal rupture	6	799 (-1257; 3061) IQR=1747	0.778
Reduction day 4 – day 7 (mU/mL)	Good outcome	80	213 (-2090; 2548) IQR=453	
	Tubal rupture	12	213 (-1467; 1150) IQR=655	0.790

* Student's t-test or Mann–Whitney test; IQR: interquartile range.

To compare cases with tubal rupture versus good outcomes in terms of β-hCG trends, a multilevel mixed linear regression model was fitted, demonstrating a statistically significant difference between groups (p < 0.001) (Figure 3).

**Figure 3:**
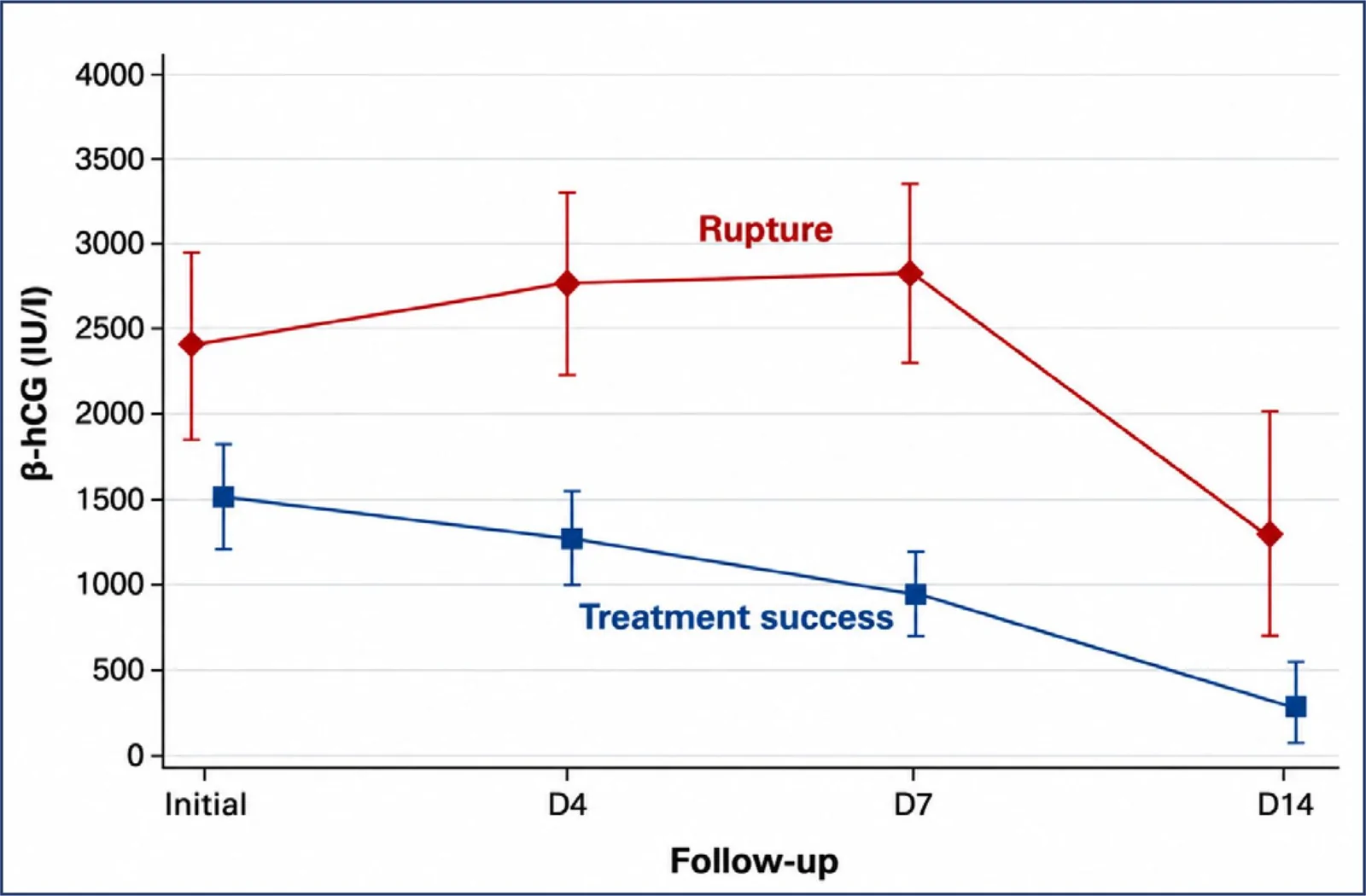
Estimated serum β-hCG values between initial and 14 days according to good and tubal rupture outcomes (p=0.001)

A second dose of methotrexate was required in 5 women with good outcomes and in 2 women who progressed to tubal rupture. There was no statistically significant association between the need for a second dose and treatment success (p = 0.664). Only one woman required hospital admission due to methotrexate-related adverse effects. She was 31 years old, primigravida, and was being treated for an 8-week tubal ectopic pregnancy, with an initial serum β-hCG level of 2,054.9 mU/mL and an adnexal mass diameter of 21 mm, allocated to the intramuscular group. After methotrexate administration, she developed dyspnea and headache, transient elevation of liver transaminases, and microcytic anemia on laboratory testing. She was hospitalized for observation, received folinic acid and symptomatic treatment, and was discharged with clinical improvement. Regarding ectopic pregnancy treatment, she had an adequate decline in serum β-hCG and a good outcome.

## Discussion

The primary objective of this randomized clinical trial was to compare the clinical efficacy and safety of intravenous versus intramuscular methotrexate for the medical management of unruptured tubal ectopic pregnancy, focusing on the binary clinical outcome of treatment success (resolution without surgery) versus failure (tubal rupture). Our findings demonstrate an overall success rate of 76.6%, with no statistically significant difference between the IV (74.6%) and IM (79.2%) administration routes.

Furthermore, adverse effects were minimal and comparable between the groups, supporting the safety of both approaches for selected hemodynamically stable patients.

While classic literature and meta-analyses often report single-dose MTX success rates between 89% and 92%^(8)^, the overall favorable outcome rate in our cohort was approximately 75%. However, a recent meta-analysis evaluating MTX treatment for tubal ectopic pregnancy reported a treatment success rate of 79.3%, which aligns closely with our overall findings.^(9)^ The discrepancy between these contemporary rates and older historical series warrants exploration. In our study protocol, expectant management was not routinely employed for asymptomatic patients with low initial β-hCG levels; instead, all eligible patients received medical treatment. Consequently, our cohort may have included a subset of patients whose pregnancies might have resolved spontaneously, potentially altering the expected response rates compared to series with highly selected populations. Additionally, the strict criteria for clinical success and follow-up in an emergency hospital setting might have contributed to a more conservative threshold for surgical intervention upon suspicion of rupture.

Furthermore, regarding the administration of a second MTX dose, our study did not demonstrate a significant association between the need for an additional dose and treatment success. This contrasts with broader literature indicating that a planned two-dose protocol may yield superior success rates compared to a strict single-dose regimen.^(5,10)^ In our study, 77% of women with favorable outcomes received a single dose of methotrexate, and the remainder required an additional dose, with no statistically significant difference between intramuscular and intravenous groups.

Known predictors of medical treatment failure include the presence of embryonic cardiac activity, larger tubal mass volume, high initial serum β-hCG concentration, the presence of free intraperitoneal fluid, and a rapid rise in β-hCG (>50% over approximately 48 hours).^(11)^ Although initial β-hCG levels and mass size are established predictors of treatment outcome, our study did not find significant differences in these baseline parameters between women with favorable evolution and those who progressed to tubal rupture. However, our secondary analysis of longitudinal β-hCG trajectories revealed that patients who achieved a successful clinical outcome exhibited a significantly more pronounced decline in β-hCG from baseline to day 4 and day 7, regardless of the administration route. This reinforces established knowledge that the initial kinetic response of β-hCG is a strong predictor of ultimate medical success.

With respect to MTX-related tissue toxicity, previous evidence suggests that toxicity is more closely related to the duration of exposure, whereas its maximal effect on rapidly dividing cells depends on the serum peak concentration, which is theoretically higher with intravenous administration.^(6)^ In a previous study by Periti et al.^(7)^, which evaluated an IV MTX protocol in 49 women without a control group, no severe adverse effects or methotrexate toxicity were reported. Our clinical results align with this safety profile. In our sample, the theoretical pharmacokinetic advantage of the IV route did not translate into a superior clinical cure rate or a faster β-hCG clearance, but it proved to be equally safe. Only one patient (in the IM group) required hospital admission due to mild adverse effects. She recovered with supportive care and folinic acid rescue, and her ectopic pregnancy resolved with adequate β-hCG decline. Overall, our findings support the safety of both routes of administration in the low-dose regimen used for tubal ectopic pregnancy.

The primary strength of this study is its randomized design, providing the highest level of evidence currently available directly comparing IV and IM MTX routes for tubal pregnancy. Nevertheless, several limitations must be acknowledged. First, missing data and losses to follow-up inherent to an emergency care setting required a per-protocol analysis, which may introduce attrition bias. Second, mild adverse effects might have been underreported due to the non-systematic nature of data collection during outpatient follow-up. Finally, the study was not initially powered as a non-inferiority trial to detect minute differences between the two routes, which should be considered when interpreting the equivalence of the clinical outcomes.

Ultimately, intravenous and intramuscular methotrexate exhibit comparable efficacy and safety profiles for the medical management of unruptured tubal pregnancy. The successful resolution of ectopic pregnancy relies on more than just the pharmacological route of isolated interventions; it requires a comprehensive, multidisciplinary clinical management strategy. The close monitoring of warning signs by the nursing staff, the psychological support necessary to navigate early pregnancy loss, and the readiness of the anesthesiology and surgical teams for emergency interventions remain the most critical components in ensuring patient safety and optimal clinical outcomes.

## Conclusion

In summary, intravenous methotrexate for the medical management of tubal ectopic pregnancy did not demonstrate superiority over intramuscular administration in terms of treatment success rates or risk of tubal rupture. The evolution of serum β-hCG levels was also similar between the two routes of administration. Adverse effects related to methotrexate did not differ meaningfully by route, supporting the equivalence of intravenous and intramuscular administration for selected hemodynamically stable patients with tubal ectopic pregnancy.

## Data Availability

The research data are described in the article presented.
